# Collusion risk in corporate networks

**DOI:** 10.1038/s41598-024-53625-9

**Published:** 2024-02-07

**Authors:** Isabela Villamil, János Kertész, Mihály Fazekas

**Affiliations:** 1 Central European University, Wien, 1100 Austria; 2 Complexity Science Hub Vienna, Wien, 1080 Austria

**Keywords:** Complex networks, Computational science

## Abstract

Collusion among economic operators increases prices, reduces product quality, and hinders innovation. Structural links can affect the incentive and ability of firms to behave competitively by facilitating collusion. We use a network-based approach to study the relationship between ownership links and bidding behavior in procurement markets. We build temporal multiplex networks based on firms’ ownership and co-bidding ties to find network measures that may signal collusion risk. We test four network measures, two at market-level (density and average harmonic closeness centrality) and two at firm-level (degree centrality and harmonic closeness centrality). Using data on public procurement contracts awarded in Sweden from 2010 to 2015, we found higher incidence of single bidding in markets that are more closely related through ownership links. Missing bidders are also more likely in these markets. Single bidding and missing bidders may indicate the presence of collusive arrangements such as of bid suppression or rotation. For the firm-level analyses, our results showed a positive relationship between winning probability and centrality in the ownership network. A similar result was obtained for cut-point position, indicating that firms that are more closely connected to other firms through ownership links have a more important position in the co-bidding network and are also more likely to win contracts.

## Introduction

While a perennial issue in competition policy, the public debate about rising corporate power has intensified in recent years. This heightened interest is due, in part, to the emergence of corporate giants in the tech industry along with the surge in ownership concentration through mergers and acquisitions. One of the foremost concerns of increased concentration is its impact on competition. The existence of structural links such as ownership overlaps, interlocking directorates, and joint ventures between firms can affect their incentive and ability to behave competitively. According to economic theory, these relationships can lead to competitive harm resulting in higher prices, poorer quality, and less innovation^1–5^. While there is substantial theoretical literature on the effects of structural links on competition, empirical studies on the subject have been limited. The renewed interest in the topic was sparked largely by two econometric papers that found that common ownership by institutional investors causes higher prices in the banking^6^ and airline^7^ industries, initiating a heated debate on whether there is a need to limit common ownership in certain markets.

Comprehensive and systematic regulation of structural links, beyond the review of mergers involving acquisitions of control, is currently absent within competition authorities. It is, in fact, still an open question whether such links truly present a competition problem that deserves closer legal scrutiny. To determine the appropriate policy response, there is thus a need for further work to show the relationship between structural links, firm behavior, and market outcomes. In this paper, we contribute to the discussion by studying the most recognized type of structural link: ownership overlaps between competitors. Specifically, we use network approaches to study the behavior of “connected” firms bidding for public procurement contracts in Sweden. There is a scarcity of empirical research specifically examining the influence of structural links on behavior and outcomes in bidding markets. However, interest in the use of new approaches to explore these complicated relationships has grown in recent years. In a working paper, Asai and Charoenwong^8^ study the effects of ownership connections on prices and cost efficiency in public procurement auctions in Singapore. Using identical bidding as an indicator for potential coordination among firms, they found the measure to be strongly correlated to having a shared owner.

**Figure 1:**
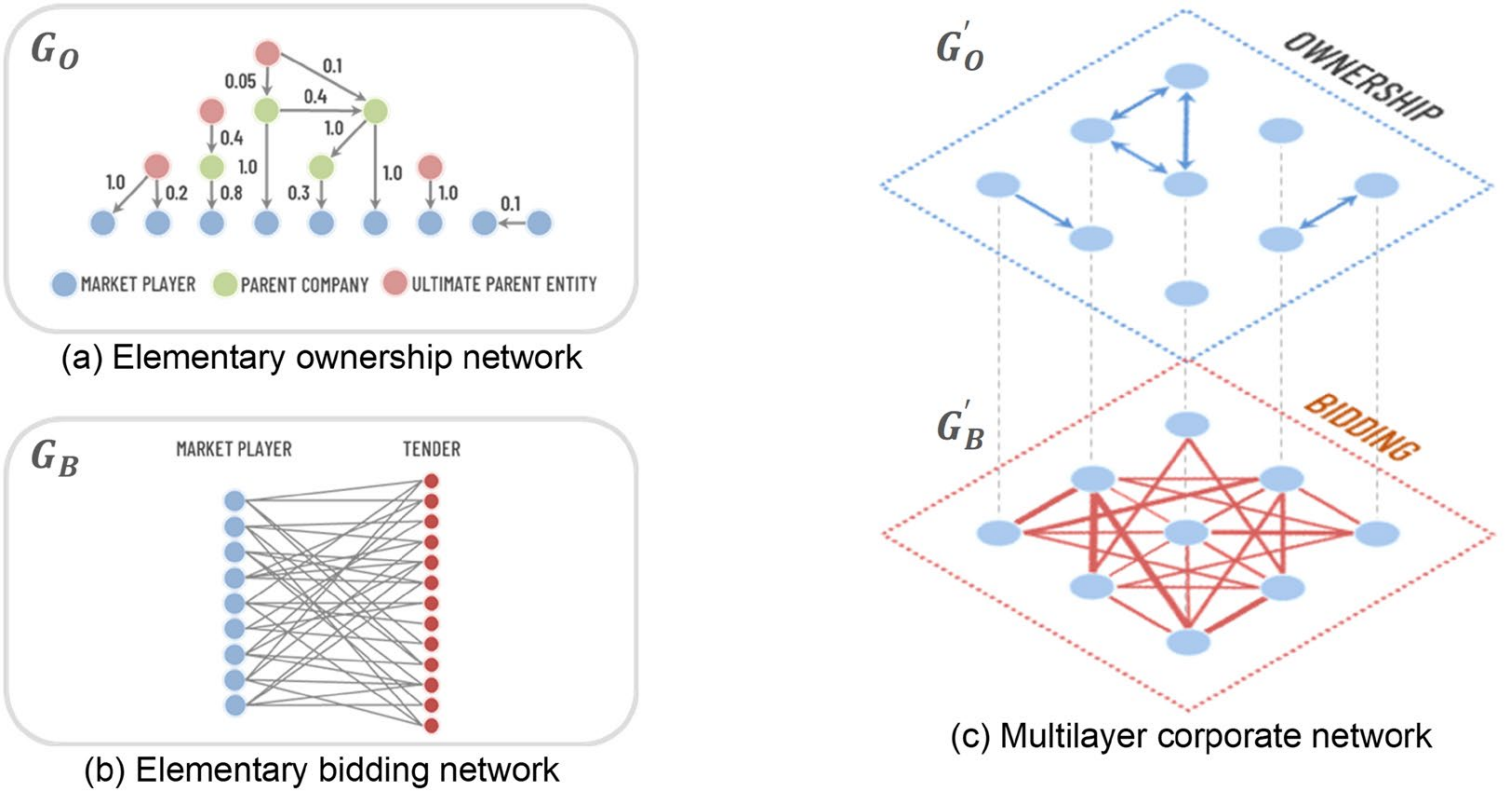
Network representation of companies. The multilayer corporate network (**c**) is constructed from the ownership network (**a**) and the bidding network (**b**).

Assessing the competitive landscape in a market is an important step in the investigations and studies conducted by competition authorities. However, complex corporate connections can make identifying relationships between firms a challenging task. Tools from network science can allow us to better measure the extent of these connections and understand how they might impact the behavior of companies that bid against each other in public procurement markets. Using dataset from Sweden, we create corporate networks consisting of 2 layers: ownership (O) and co-bidding (B). A visual representation of the multilayer network is provided in Fig. 1c. The undirected weighted network layer $G_{B}^\prime$ is a projection of the bipartite graph $G_{B}$, which connects firms with tenders, as shown in Fig. 1b. In $G_{B}^\prime$, each edge has an associated weight $w_{ij,M}^{[B]}$ indicating the number of tenders where both firms *i* and *j* submitted a bid.

The undirected market ownership network $G_{O}^\prime$ is also a projection; in this case of what we call the elementary ownership network $G_{O}$, illustrated in Fig. 1a. In $G_{O}$, the vertices correspond to the economic entities, and the links to the ownership shares connecting them. Building $G_{O}$ involves creating a network of relationships among firms, tracing back to the level of the ultimate parent entity, if available. An edge $w_{ij,M}^{[O]}$ in $G_{O}^\prime$ indicates the existence of an ownership link between competitors *i* and *j*.

Our analysis focuses on the relationship between ownership links and competitive behavior. Collusion requires firms to reach an understanding on the terms of coordination and to be able to monitor these terms, allowing deviations to be detected and punished. Ownership links can facilitate this by leading to or increasing information exchange and transparency between competitors. However, the relationship between collusion and bidding can be positive or negative, depending on the type of bid-rigging arrangement. For instance, cover bidding and bid rotation can result in more co-bidding between cartel members, while bid suppression and market allocation will lead to less bids submitted by firms.

We begin our empirical analysis by looking at pair-level data to determine if there is a correlation between the presence of an ownership tie and the frequency of co-bidding. We then explore the theory that structural links weaken competition by relating ownership network measures with collusion risk indicators. There is extensive literature centering on the detection of collusive behavior through the analysis of patterns in bidding or pricing^9–14^. In this paper, we use five indicators of collusion risk in public procurement. Three of these are calculated at the market level (rate of single bidding, number of missing bidders, and stability of market shares), while the other two are firm level (winning probability and cut-point position).

The most straightforward way to remove competition is through suppressing bids, hence high rates of single biding or the absence of bids from previously active companies may indicate collusive behavior. The winning probability of firms is another indicator that can be used in identifying a strategy based on withholding bids as well arrangements that involve cover bidding. Cover bidding refers to the practice of submitting intentionally losing bids, with the aim of creating an appearance of competition while ensuring that a pre-selected firm wins the contract. Companies with very high winning probabilities may be a sign of a competitive anomaly.

A market structure that is overly stable, as measured by low variation in the market shares of firms, can also suggest collusion risk. In certain bid-rigging arrangements, firms may agree to maintain their market positions by allocating contracts among themselves or engaging in bid rotation to avoid disruptive competition. Finally, the position of a company in a market can provide information on the competitive environment. A network-based approach to assess the importance of market players has been suggested by Tóth et al.^15^, which involves identifying firms that are in a cut-point position in the co-bidding networks. Cut-points are crucial nodes that connect otherwise unrelated nodes. In the context of co-bidding, companies in cut-point positions are those that extensively bid against numerous competitors who may not interact with each other. The presence of a company in a cut-point position may indicates its role as a connecting element in a collusive arrangement, particularly those involving the submission of cover bids.

## Results

### Pairwise correlation

**Figure 2:**
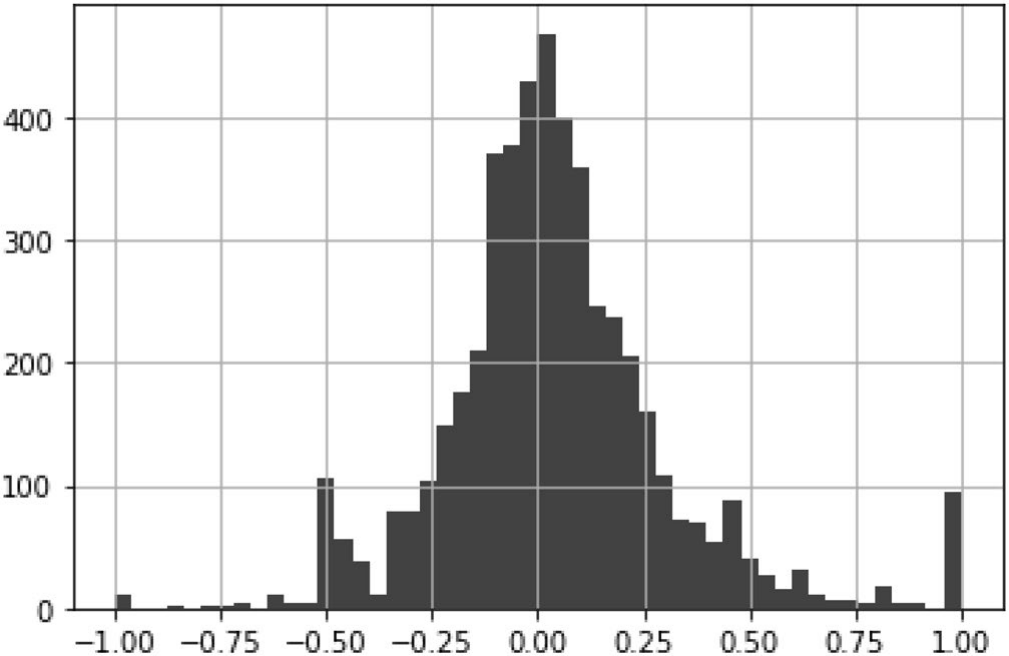
Distribution of Pearson point biserial correlation coefficients per market. The correlation between ownership and co-bidding differs across markets.

We use pair-level data to investigate if the presence of an ownership tie relates to the co-bidding behavior of firms. For each market *M*, we calculate the point bi-serial correlation coefficient between the variables $w_{ij,M}^{[B]}$ (number of co-bids) and $w_{ij,M}^{[O]}$ (ownership link). The frequency distribution of these coefficients for all markets is shown in Fig. 2. We see that the correlation between ownership and co-bidding differs across markets. In some cases, the relationship is negative while positive in others. Markets in the extreme sides with outlier values may indicate the presence of suspicious behavior. Indeed, dividing our dataset into quartiles based on market size, we find that higher negative and positive correlations are more common in smaller markets with less than 9 firms. Based on economic theory, we expect cartels to be more stable when there are fewer firms competing in the market.

**Figure 3:**
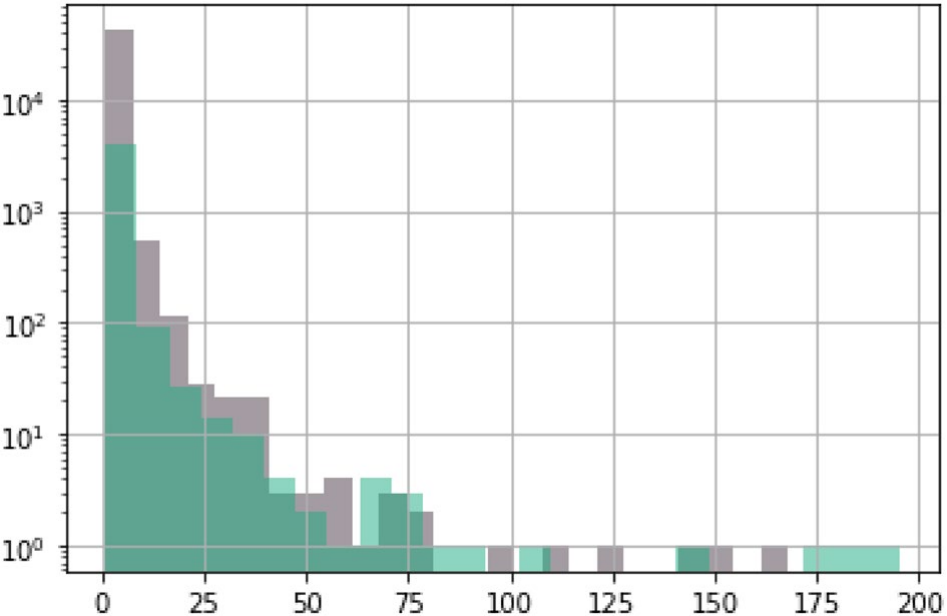
Distribution of number of co-bids between firms that have no ownership link (gray) and firms that have ownership link (green).

**Figure 4:**
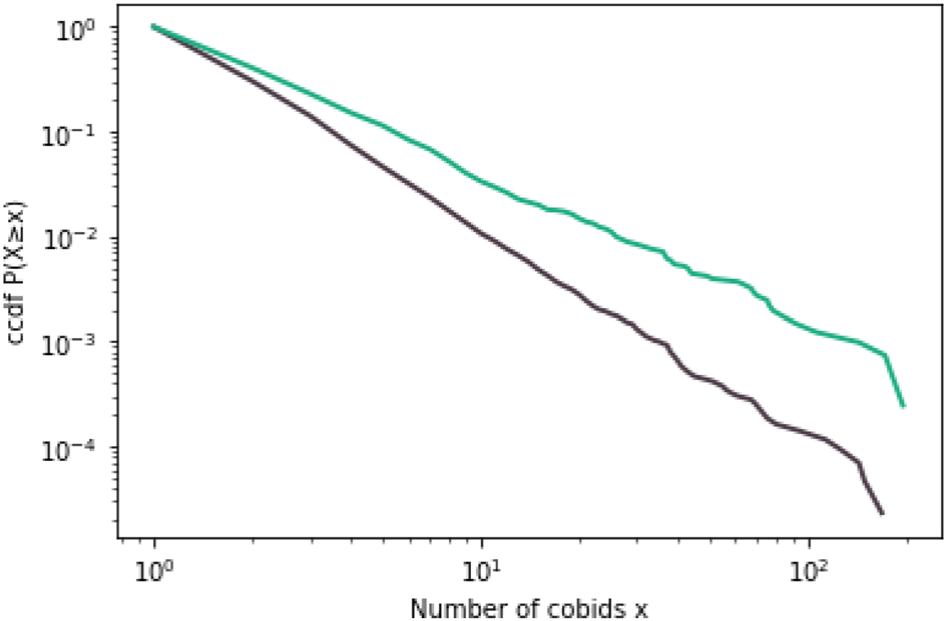
Complementary cumulative distribution of co-bidding for firms that have no ownership link (gray) and firms that have ownership link (green).

We explore the robustness of these results further by looking at the relationship between co-bidding and ownership ties without regard for market definition. This approach has the benefit of also capturing multi-market contact, which can likewise facilitate collusion. We compare the distribution of the number of co-bids between firms with ownership links versus firms with no ownership link and find a significant difference between the two (see Figs. 3 and 4). We find that similar to the results from the market-level correlations, co-bids appear to be higher between firms with ownership link. The exact reason for such behavior is uncertain. While such behavior is consistent with bid rigging agreements such as cover bidding and bid rotation, the relationship could also be due to other more innocuous factors. It is, for instance, possible that firms with ownership links are more likely to receive the same information on bidding opportunities. Another possibility is that firms with ownership links are larger and therefore submit more bids, increasing the likelihood of co-bidding with any firm. In fact, we found that on average firms with no ownership link submit bids for only 3 tenders in a year, while the average for those with at least one ownership link is 7.

### Regression analyses

Our succeeding analyses explores the relationship between ownership network structure and collusion risk. Recognizing that ownership links can act as a facilitating force for collusion in public procurement markets, we put forth the following hypothesis: (i) markets with more ownership links have greater risk of collusion, as measured by the incidence of single bidding, the proportion of missing bidders, and the stability of market shares; and (2) firms with more ownership links have higher probability of winning and are more likely to be in a cut-point position in the bidding network.

We focus on three ownership network measures: density, harmonic closeness centrality, and degree centrality. We fit five regression models on our dataset, one for each collusion risk indicator. In each model the key explanatory variable is the ownership network measure. Four of our dependent variables are continuous: single bidding, missing bidders, winning probability, and stability. For these variables we fit linear regression models. On the other hand, the dependent variable cut-point position is a binary outcome. We therefore model this outcome using a logistic regression.

**Figure 5:**
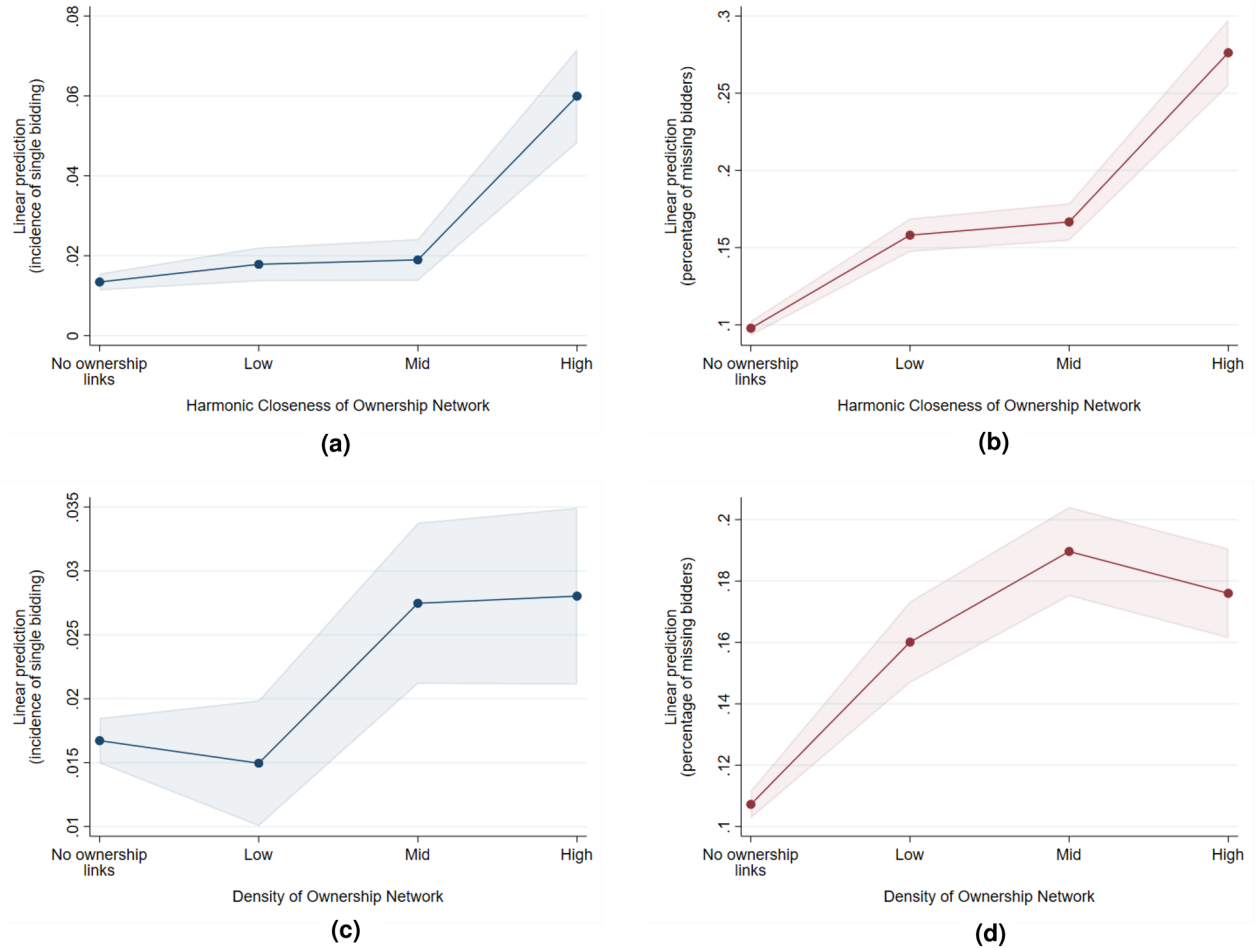
Predictive margins of market-level indicators (with 95 percent confidence intervals). In general, collusion risk is lower when there are no ownership links. However, when links are present, the relationship is not necessarily monotonic.

**Figure 6:**
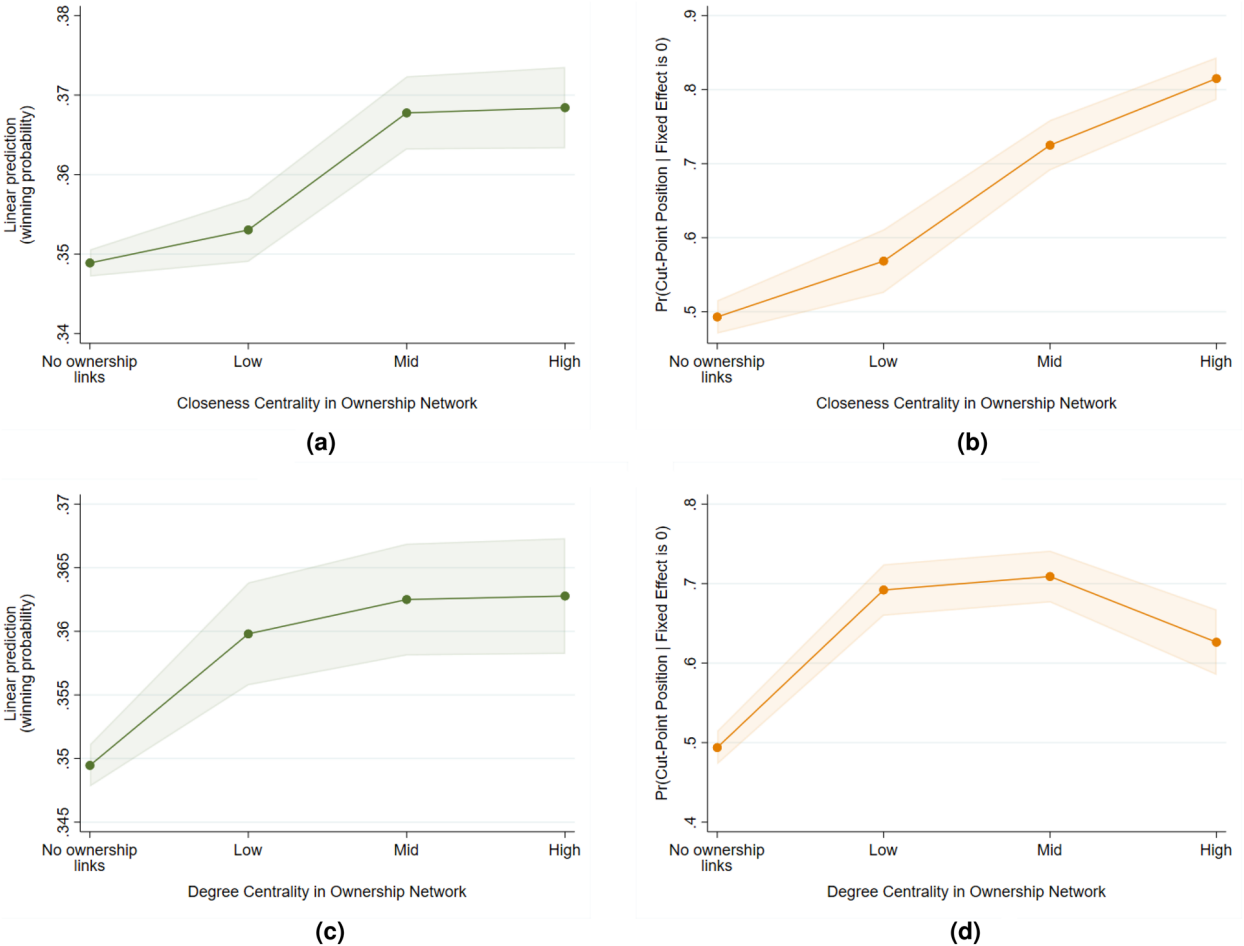
Predictive margins of firm-level indicators (with 95 percent confidence intervals). In general, collusion risk is lower when there are no ownership links. However, the relationship between the number of ownership links and the collusion risk measures is not always increasing.

The results of our regressions are summarized in Table 1 (more detailed tables are available in the Supplementary Materials). In addition, we attempt an alternative specification where the explanatory variables are inputted as categories instead of a linear relationship. Specifically, we divide the observations into quantiles, indicating varying levels of ownership links (none, low, mid, high) based on the value of the network descriptors. Higher values of the category indicate more ownership links. The resulting predictive margins are plotted in Figs. 5a to 5d and 6a to 6d. In general, we find that collusion risk is lower when there are no ownership links at all. However, when links do exist, higher values of the measures do not necessarily predict higher incidence of collusive behavior. For instance, we find that markets with high-density ownership networks actually have less missing bidders compared to mid-density networks. In contrast, networks with high average harmonic closeness centrality have more single bidding and missing bidders compared to low and mid.

**Table 1:** Summary of regression results. Regression coefficients and standard errors (in parentheses). The positive values indicate a positive relationship between ownership ties and collusion risk.

Group	Market-level ownership structure indicators		Firm-level ownership structure indicators	
Bidding pattern				
Indicator name	Harmonic closeness (ave)	Density	Harmonic closeness	Degree
Single bidding	0.0048** (0.0022)	0.0705*** (0.0212)	–	–
Missing bidders	0.0420*** (0.0097)	0.1423*** (0.0253)	–	–
Winning probability	–	–	0.0007*** (0.0001)	0.0171*** (0.0047)
Cut-point position	–	–	0.0590*** (0.0047)	− 0.1288 (0.1002)
Market structure				
Stable market structure	-0.0002 (0.0018)	-0.0268 (0.0211)	–	–

*$$p<0.10$$, **$$p<0.05$$, ***$$p<0.01$$

Our first market-level indicator is single bidding, defined as the proportion of tenders that receive only one bid. In the presence of bid suppression, we expect the incidence of single bidding to be larger. We find that the relationship between proportion of single bidding is positive for both average harmonic closeness centrality and density. Higher values of closeness centrality indicate shorter paths between firms. We therefore find that shorter paths in the ownership network, which signal higher potential for collusion, is associated with a higher proportion of single bidding. This relationship is consistent with behavior in bid suppression, as colluding firms submit less bids to avoid competing with each other. Note that the effect of network density is the same—markets with more dense ownership structure have higher rate of single bidding compared to those with low or no ownership links.

Our next indicator is missing bidders, which is based on the number of bids submitted. It is a less extreme version of single bidding, indicating the proportion of competitors that did not a submit a bid for a tender. A high value of the indicator can indicate that a bid suppression scheme is operating in the market; however, a low value may signal cover bidding. We find that the relationship between proportion of missing bidders and harmonic closeness centrality is positive, and that the same is true with density. As discussed above, markets with higher average centrality are expected to have less intense competition due to easier coordination and faster information flow between companies. Our results indicate that in markets with more firms that are more closely linked through ownership, the average percentage of missing bidders is higher. This finding is consistent with our result from single bidding–in markets where collusion risk due to ownership links is higher, less firms consistently submit bids.

However, based on the predictive margins shown in Fig. 5c and d, we can also observe that the effect of network density on collusion risk can actually be decreasing between certain levels. This indicates that the relationship is not linear and that very high values of ownership network density may actually be associated with less single bidding and less missing bidders. In contrast, very high values of harmonic closeness centrality coincides with more single bidding and more missing bidders (see Fig. 5a,b). These results suggest an interesting finding—the topology of the links (who is connected to whom) matters more than the number of links between firms in the ownership network.

The firm-level indicator winning probability is defined as the share of contracts a company has won out of all the bids they submitted for a given relevant market. Our interest lies in the relationship between winning probability and firm-level ownership network measures; specifically, the degree (i.e., the number of other firms it shares an ownership link with) and the harmonic closeness centrality. The predicted effect of ownership connections on the winning probability of a firm is ambiguous. A collusive ring engaged in cover bidding can lead to some firms having artificially high winning rates while those participants that submit a larger number of courtesy bids may have artificially low winning rates. On the other hand, in the case of bid suppression, we expect firms with more ownership links to have higher winning probability due to less actual competition. Our results show that firms with more ownership connections have higher winning probability, which again is consistent with the presence of bid suppression. However, the relationship is not necessarily linear as firms with very high values of degree and harmonic closeness centrality have similar winning probability to those with mid levels (see Fig. 6a,c). These firms with high ownership links but low winning probability may warrant closer scrutiny as they may be the ones more likely to submit cover bids.

Our next indicator is cut-point position. A company is defined to be in a cut-point position if its removal from the co-bidding network increases the number of connected components of the graph. A firm with a cut-point position may more be more likely to act as a ringleader or to take on a central role in the coordination and formation of a cartel. We use a binary indicator to define if a firm is in a cut-point position and use a logistic model for this regression. Consistent with expectation, we find a positive relationship between cut-point position and harmonic closeness centrality. Our results thus indicate that firms that are more easily able to reach other firms through ownership links are more likely to be in a cut-point position in the co-bidding network, emphasizing their importance. While the coefficient for degree centrality is not significant in our linear specification, we do find that firms with no ownership links have significantly lower probability of being in a cut-point position compared to those that have at least one tie (see Fig. 6b,d). To detect the presence of core actors in a collusive network, the harmonic closeness centrality may therefore be a suitable indicator.

Our last indicator is market share stability, which can signal collusive behavior under certain conditions. We define stability as one minus the average absolute change in market shares from the previous year. The lower the value, the less stable the market. Since there is insufficient data on the value of contracts, we used the share of the number of tenders won by each firm. Markets with bid-rigging cartels are expected to have more stable market shares. We therefore expect the relationship between this indicator and our ownership network indicators to be positive. A weakness of the stability indicator is that it is unable to capture partial cartels. If not all active firms in a relevant market participate in the collusive arrangement, then market shares may fluctuate as the number of bids won by these firms changes^16^. Indeed, our results show that while there is a negative relationship between stability and our ownership network descriptors, this relationship is not significant when we control for the size of the relevant market.

## Discussion

Our study provides groundwork for future research on the competitive effects of structural links in procurement markets. We find that in markets with firms that are more closely related through ownership links, there is higher incidence of single bidding and more missing bidders. However, the relationship between ownership network density and missing bidders is not linear, suggesting that the structure of the relationships between firms matters more than just the number of links. We also find that ownership indicators have no significant relationship with the stability of market shares. This may be due to the weakness of the indicator as a measure of collusion. For our firm-level analyses, we find a positive relationship between cut-point position and harmonic closeness centrality. This shows that firms that are more closely linked to others also hold an important position in the co-bidding network. Winning probability also has a positive relationship. In the presence of bid suppression with less competing firms, this result is not unexpected.

These results support the argument that there is a need for greater vigilance in monitoring the behavior of firms that have ownership links. While we make no claims of causality, the positive relationship between ownership links and collusion risk does suggest that firms with such connections may be more prone to engage in anti-competitive behavior. Our findings also highlight the importance of transparency in ownership and control structures. An important topic that has become central to global efforts to address money laundering is the issue of beneficial ownership. Many argue that the lack of transparency and identification of individuals who ultimately own or control a company creates opportunities for illicit activities, including tax evasion and corruption. By hiding behind layers of complex ownership structures, beneficial owners can obscure their true identities and intentions, making it difficult for regulators, law enforcement agencies, and the public to hold them accountable. Establishing robust reporting and disclosure mechanisms that compel companies to reveal their beneficial owners will enable regulators and competition authorities to better identify and investigate potential conflicts of interest, related-party transactions, and collusive arrangements.

There are multiple avenues for future work. One the one hand, the methodology can be refined using the same data, while on the other hand, the approach can be expanded to incorporate more data and other contexts. First, our approach using correlations and regressions could only identify the average effects and general relationships. However, it is quite possible that collusion in Sweden is more of an outlier behavior rather than the norm. Further work could depart from the regression results and instead look at extreme or outlier cases where the hypothesized relationships are the strongest. This would mean to flag cases with the densest ownership ties and collate them with the highest value cartel screens (i.e., highest cartel risks). In addition, convergence among different ownership and competitive behavior-based indicators should not be bivariate because cartels are most likely to leave multiple markers at the same time. This implies that on top of looking at bivariate relationships such as ownership density and single bidding, it could further strengthen measurement validity to look at sets of indicators corresponding to the same cartel type. This could be done, for example, by looking at the highest risk markets indicator by indicator until a subset of markets of companies are identified where are relevant cartel screens point at cartel presence.

Second, a natural next step is to expand the scope of the investigation by including data from other countries and conducting cross-country comparisons. This would also allow us to test other collusion risk indicators that use price data, which is available for certain countries. Another valuable addition to this work would be to include a measure of ownership stakes between firms. With this information we can distinguish among the types of ownership links, enabling more sophisticated analysis.

Lastly, we suggest studying other types of structural links. Our focus so far has been on ownership; however, other connections between firms-such as director interlocks, joint bidding agreements, and subcontracting-may likewise have anti-competitive effects. These links also provide the possibility for firms to control or influence the behavior of their competitors, aside from providing a channel to transfer information that can enhance the stability of a collusive equilibrium. Such research, identifying and characterizing the complex interactions between firms and investigating their impact on competition, can help guide discussions of policy responses to structural links.

## Data and methods

### Data used

We use two large-scale administrative datasets based on official government sources. First, we build on public procurement data collected by the Government Transparency Institute (GTI) from government advertisement portals. Second, we rely on the Orbis Global Database from the Bureau van Dijk collected from government company registers. The scope of our analysis is limited to Sweden in the period 2010-2015 where we have sufficiently high-quality overlapping data on ownership and tenders. We use product codes in the public procurement data (the so-called Common Procurement Vocabulary or CPV codes) to categorize tenders into product groups. We then identify which firms submitted bids for these tenders to obtain the set of firms that compete in that market.

Our dataset is restricted by the number of firms in the GTI database that we can match to the Orbis database. After removing observations that we are unable to match, we are left with 170,442 data points in our bid-level procurement data, with 6909 distinct bidders and 50,598 distinct tenders. For each year, we were able to identify between 1694 and 2005 relevant markets that have at least two active firms. The majority of these markets are small, with more than half having less than nine firms. This observation is depicted in the histogram shown in Fig. S1. The Supplementary Materials also includes Table S1, which details the annual count of observations, and Table S2, which provides mean values of the collusion risk indicators. The latter reveals that markets and firms with ownership links generally exhibit higher levels of collusion risk.

### Market definition

To construct the corporate networks, we must first define the “relevant markets”—the catalogue of goods or services that are considered substitutable by consumers. In practice, defining a relevant market often calls for detailed economic analyses. The standard framework employed by competition authorities is the test of small but significant and non-transitory price increases, which seeks to identify the smallest market within which a hypothetical monopolist or cartel could impose a profitable significant increase in price. In this paper, we take a simplified approach and adopt the method suggested by Fazekas and Tóth^17^, which uses product and firm attributes. Specifically, we identify the product groups based on the Common Procurement Vocabulary (CPV) categories and the geographical location based on the Nomenclature of Territorial Units for Statistics (NUTS) of the contracting authority. This allows us to determine the groups of firms that we can consider as competitors.

### Network construction

**Figure 7:**
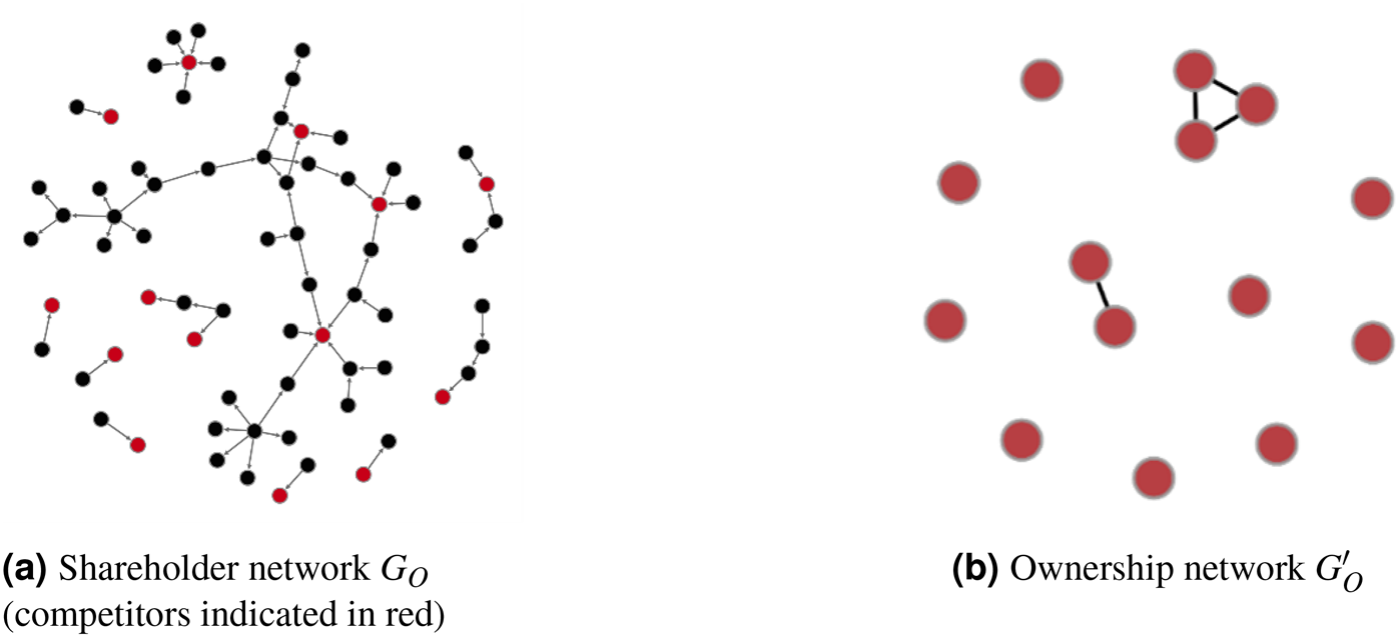
Illustration of a shareholder network (**a**) and the corresponding ownership network (**b**). Nodes in the ownership network $G_{O}^\prime$ represent firms competing in a market. An edge indicates the presences of an ownership link.

For each relevant market, we create a multi-layer corporate network containing information on ownership (O) and co-bidding (B). To build the elementary ownership network $G_{O}$, we conduct a recursive exploration of the neighborhood of these companies in the Orbis database: first proceeding upstream with a breadth-first search to identify all direct and indirect shareholders of the market participants, and then continuing in a similar way downstream to identify companies that are directly and indirectly owned by the competing firms. Each ownership network $G_{O}^\prime$ is created from the projection of $G_{O}$, reducing the nodes to only those identified as competitors based on the GTI data. To illustrate this process, we provide an example with a market consisting of 14 competitors, as shown in Fig. 7—$G_{O}$ contains bidders and their shareholders, while $G_{O}^\prime$ shows only bidders and edges indicating ownership links.

We have chosen to mark competitors *i* and *j* as having an ownership link regardless of the number of steps it takes to reach *i* from *j* in the elementary ownership network. An alternative approach taken by Asai and Charoenwong^8^ is to only consider connections between firms up to a certain level. However, using such a cut-off may lead us to exclude links that may still be meaningful, especially if the shares held by the common owner is significant.

Building the co-bidding networks is more straightforward. Using our procurement data, we count the number of times each pair of firms bid on the same tender in a market. We can then analyze these networks together to study their relationships.

In this analysis, we make no distinction between the types of ownership overlaps (e.g., one-sided versus cross-ownership, partial versus full control). $w_{ij,M}^{[O]}$ is therefore a binary variable that takes on the value 1 when an ownership link is present, and 0 otherwise. Admittedly, this approach disregards valuable information that may have important implications on the competitive environment in a market. Indeed, Gilo et al.^18^ and Shelegia & Spiegel^19^ show how asymmetric stakes in rivals can have differing effects on the behavior of firms. To capture information on the magnitude of ownership linkage, it is possible to associate a weight with each edge $w_{ij,M}^{[O]}$ that measures the effective stake of firm *i* in *j*. This will transform $G_{O}^\prime$ into a directed weighted network, which we can then analyze and compare against our other network layers. Such an approach is suggested for future work.

### Empirical analysis

We begin our empirical analysis by looking at pair-level data to determine if there is a correlation between the presence of an ownership tie and the frequency of co-bidding. We then estimate how various collusion risk indicators (single bidding, missing bidders, winning probability, cut-point position, and stability) relate to different measures that describe the connectivity of the ownership networks (density, harmonic closeness centrality, and degree centrality). In our regressions, we use a panel model with fixed effects and robust standard errors. We use the Hausman test to confirm that the fixed effects specification is more appropriate than random effects, and the modified Wald test to check for groupwise heteroskedasticity.

For our market-level collusion risk indicators—single bidding, missing bidders, stability—, the model is specified as follows:

$$y_{mt} = \beta _0 + \beta _1 x_{mt} + \beta _2 {\textbf {z}}_{mt} + \alpha _m + \gamma _t + \varepsilon _{mt},$$

where $y_{mt}$ is the value of the collusion risk indicator for market *m* in year *t*, $x_{mt}$ is the value of the ownership network measure of interest, and ${\textbf {z}}_{mt}$ is a vector of variables controlling for the size of the market and the number of firms that have an ownership link. $\alpha _m$ represents the fixed effects for each market *m*, capturing time-invariant heterogeneity at the market level, and $\gamma _t$ represents the fixed effects for each time period *t*, capturing time-specific factors.

For firm-level collusion risk indicators—winning probability and cut-point position—, the regression models are specified on the firm-level:

$$y_{ft} = \beta _0 + \beta _1 x_{ft} + \alpha _f + \gamma _t + \varepsilon _{ft},$$

where $y_{ft}$ is the value of the collusion risk indicator for firm *f* in time period *t*, and $x_{ft}$ is the value of the firm-level ownership network measure. In the case of cut-point position, we use a conditional logit model since $y_{ft}$ is a binary indicator. For both regressions we include period fixed effects ($\gamma _t$) to control for common time shocks as well as firm fixed effects ($\alpha _m$), to control for time-invariant heterogeneity at the firm level. This provides more robust estimates of the relationships with our independent variables.

In addition to these, we implement an alternative specification for each regression where the explanatory variables are entered as categorical variables rather than continuous variables to test potential non-linear relationships. We divide the observations into quantiles, indicating varying levels of ownership links—none, low, mid, or high. We then compute for the predictive margins and compare the values of collusion risk for these different levels. It is important to note that our regression models have limitations in accounting for endogeneity arising from unobserved factors or reverse causality, which restricts us from making causal claims. Nevertheless, our analysis offers valuable insights into the relationship between ownership links and anti-competitive behavior.

Our study focuses on three ownership network measures: density, harmonic closeness centrality, and degree centrality. Density captures the interconnectivity of firms in the network and is computed as $D=\frac{2\left| E\right| }{\left| V\right| \left( \left| V\right| -1\right) }$, where $\left| V\right|$ and $\left| E\right|$ denote the number of vertices and edges, respectively. It may vary from low density, where a group of firms are loosely connected, to high density, where firms are highly interlinked. Markets with higher ownership network density are expected to have less intense competition due to shared knowledge, better information transmission, and higher trust between firms. Therefore, if colluding firms engage in bid suppression or rotation, we expect markets with high ownership network density to have greater incidence of single bidding and larger proportion of missing bidders. In addition, the market structure is expected to be relatively more stable.

Closeness centrality captures the average length of the shortest path between firms in the ownership network, and in its standard form is computed as $C_{i}=\frac{1}{\sum _{j\ne i}dist\left( i,j\right) }$, where $dist\left( i,j\right)$ is the distance between nodes *i* and *j*. However, since ownership networks are rarely fully connected, there may be cases where there are no paths connecting nodes, resulting in infinitely large centrality scores. We thus instead use the harmonic closeness centrality, which is computed as $\sum _{j\ne i}\frac{1}{dist\left( i,j\right) }$. This value measures how close a node is to the other nodes in a network. In social network analysis, firms with higher closeness centrality are often considered important due to their access to greater and quicker information and research exchange. We test this indicator in both our firm-level and market-level regressions, using the average value across firms for the latter. Similar to density, markets with higher closeness centrality are expected to have less intense competition due to easier coordination and faster communication. The expected relationship with single bidding, missing bidders, and stable market structure is therefore the same. At the firm-level, we expect companies with high closeness centrality to be more likely to be in a cut-position in the co-bidding network. The effect on winning probability is however ambiguous, depending on whether the colluding firm submits more cover bids than genuine bids.

Degree centrality captures the local connections of firms. The degree centrality of a node *i* is defined simply as the number of links incident upon that node. We use normalized values by dividing this number by the maximum possible degree $\left| V\right| -1$. Firms with high degree centrality are considered to be important actors due to their direct connections with other firms through ownership links, allowing easier communication and greater possibility to influence or coordinate behavior. Since the measure is a stricter version of closeness centrality, its relationship with our collusion risk indicators should be the same.

### Supplementary Information

Supplementary Information.

## Data Availability

The datasets generated during the study have been anonymised according to the conditions of the data providers. The aggregated data are available for download at: https://www.dropbox.com/sh/j2phr3x7s1nac1m/AABOdkd9VYwhnKAlFEd6coQJa?dl=0.
