# Supplementary material for: Collusion risk in corporate networks
Source: Sci Rep. 2024 Feb 7;14:3161. doi: 10.1038/s41598-024-53625-9 (PMC10850496; doi:10.1038/s41598-024-53625-9)
Supplement: Supplementary file 1 — Supplementary Information. [file 41598_2024_53625_MOESM1_ESM.pdf]

Supplementary Materials

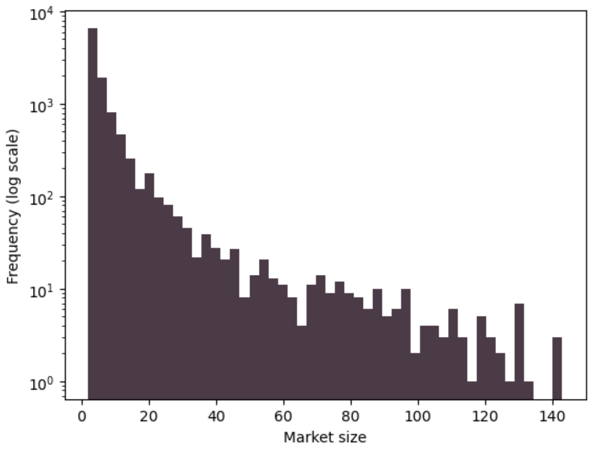

Figure S1. Distribution of market sizes

Table S1. Count of observations per year

	Number of distinct markets	Number of distinct firms	Number of firm-level observations*
2010	1,694	3,794	12,813
2011	1,774	4,315	13,135
2012	1,956	5,339	17,376
2013	2,005	5,451	17,954
2014	1,840	5,127	16,155
2015	1,746	5,033	15,347

*Firms can be active in multiple markets. On average, firms submit bids to 3 different markets.

Table S2. Mean values of collusion risk indicators

	All markets	Markets with at least one ownership link	Markets with no ownership link
Percentage of single bids (mean)	0.0190	0.0298	0.0129
Percentage of missing bidders (mean)	0.1313	0.2695	0.0537
Market share stability (mean)	0.9471	0.9605	0.9365
	All firms	Firms with at least one ownership link	Firms with no ownership link
Winning probability (mean)	0.3534	0.3881	0.3367
Cut-point position (proportion)	0.0520	0.0788	0.0391

Table S3. Results of market-level regressions (single bidding and missing bidders)

	(1) Single bidding	(2) Single bidding	(3) Missing bidders	(4) Missing bidders
Harmonic closeness centrality (ave)	0.0048**		0.0420***	
	(0.0022)		(0.0097)	
Density		0.0705***		0.1423***
		(0.0212)		(0.0253)
Num of connected firms	0.0002	−0.0003	−0.0007	0.0056**
	(0.0006)	(0.0005)	(0.0024)	(0.0022)
MarketSize	−0.0001	0.0003	0.0064***	0.0062***
	(0.0002)	(0.0002)	(0.0007)	(0.0008)
Year=2010	0.0000	0.0000	0.0000	0.0000
	(.)	(.)	(.)	(.)
Year=2011	−0.0016	−0.0016	−0.0009	−0.0019
	(0.0037)	(0.0036)	(0.0063)	(0.0063)
Year=2012	0.0012	0.0010	0.0028	0.0020
	(0.0033)	(0.0033)	(0.0065)	(0.0065)
Year=2013	0.0016	0.0013	0.0002	−0.0021
	(0.0034)	(0.0034)	(0.0064)	(0.0064)
Year=2014	0.0038	0.0037	0.0041	0.0017
	(0.0035)	(0.0035)	(0.0065)	(0.0065)
Year=2015	−0.0015	−0.0026	−0.0084	−0.0112*
	(0.0034)	(0.0034)	(0.0063)	(0.0062)
Constant	0.0174***	0.0136***	0.0712***	0.0697***
	(0.0025)	(0.0027)	(0.0060)	(0.0056)
Market FE	*Yes*	*Yes*	*Yes*	*Yes*
Observations	11015.0000	11015.0000	11015.0000	11015.0000
R^2	0.0025	0.0076	0.1492	0.1403

Standard errors in parentheses

* $p < 0.10$, ** $p < 0.05$, *** $p < 0.01$

Table S4. Results of market-level regressions (stability)

	(1)<br>Stability	(2)<br>Stability
Harmonic closeness centrality (ave)	−0.0002	
	(0.0018)	
Density		−0.0268
		(0.0211)
Num of connected firms	−0.0006	−0.0002
	(0.0009)	(0.0007)
MarketSize	0.0011***	0.0009***
	(0.0002)	(0.0002)
Year=2011	0.0000	0.0000
	(.)	(.)
Year=2012	−0.0025	−0.0023
	(0.0042)	(0.0042)
Year=2013	0.0004	0.0004
	(0.0039)	(0.0039)
Year=2014	−0.0026	−0.0026
	(0.0040)	(0.0040)
Year=2015	−0.0123***	−0.0120***
	(0.0043)	(0.0043)
Constant	0.9414***	0.9430***
	(0.0026)	(0.0029)
Market FE	*Yes*	*Yes*
Observations	5861.0000	5861.0000
R^2	0.0103	0.0110

Standard errors in parentheses

* $p < 0.10$, ** $p < 0.05$, *** $p < 0.01$

Table S5. Results of firm-level regressions

	(1) Winning probability	(2) Winning probability	(3) Cut-point position	(4) Cut-point position
main				
Harmonic closeness centrality	0.0007***		0.0590***	
	(0.0001)		(0.0047)	
Degree centrality		0.0171***		−0.1288
		(0.0047)		(0.1002)
Year=2010	0.0000	0.0000	0.0000	0.0000
	(.)	(.)	(.)	(.)
Year=2011	0.0236***	0.0236***	0.0059	−0.0271
	(0.0049)	(0.0049)	(0.0584)	(0.0578)
Year=2012	0.0172***	0.0175***	0.0104	0.0305
	(0.0047)	(0.0047)	(0.0552)	(0.0531)
Year=2013	0.0094**	0.0096**	−0.0672	−0.0631
	(0.0048)	(0.0048)	(0.0633)	(0.0599)
Year=2014	0.0176***	0.0178***	0.0483	0.0446
	(0.0053)	(0.0053)	(0.0673)	(0.0638)
Year=2015	0.0215***	0.0217***	0.1001	0.1247**
	(0.0054)	(0.0054)	(0.0660)	(0.0633)
Constant	0.3371***	0.3369***		
	(0.0036)	(0.0036)		
Firm FE	*Yes*	*Yes*	*Yes*	*Yes*
Observations	92780.0000	92780.0000	49992.0000	49992.0000
R^2	0.0023	0.0022		
Pseudo-R^2			0.0139	0.0006

Standard errors in parentheses

* $p < 0.10$, ** $p < 0.05$, *** $p < 0.01$
